# Environmental risk factors are associated with autoimmune hepatitis

**DOI:** 10.1111/liv.14944

**Published:** 2021-05-28

**Authors:** Craig Lammert, Sai N. Chalasani, Elizabeth J. Atkinson, Bryan M. McCauley, Konstantinos N. Lazaridis

**Affiliations:** 1 Division of Gastroenterology and Hepatology, Indiana University School of Medicine, Indianapolis, IN, USA; 2 Division of Gastroenterology and Hepatology, Mayo Clinic College of Medicine, Rochester, MN, USA

**Keywords:** autoimmune hepatitis, environment, infection, oestrogen, UTI, vaccine

## Abstract

**Background:**

Failure of immunologic homeostasis and resultant hepatocyte destruction in autoimmune hepatitis (AIH) is likely the result of environmental triggers within a permissive genetic architecture.

**Aims:**

We aimed to identify risk factors associated with AIH in a well-phenotyped AIH cohort.

**Methods:**

We prospectively collected environmental questionnaires from 358 AIH cases and 563 healthy controls. Response frequencies were compared using logistic regression, adjusting for age at recruitment, sex and education.

**Results:**

AIH cases were more likely to ever have a urinary tract infection (UTI) (53.6% vs 33.9%, *P* < .001) and recurrent UTI (more than 1 per year) (23.5% vs 15.9%, *P* = .002) compared to controls. Female cases more frequently had ever used oral contraceptives (83.0% vs 73.7%, *P* = .006), fewer pregnancies (median = 1 vs 3, *P* < .001) and less often used hormone replacement therapy compared to controls (28.5% vs 60.1%, *P* < .001). Current smoking was more prevalent in cases (18.9% vs 7.4%, *P* = .022), yet no difference according to historical smoking behaviours was observed. Finally, cases were less likely to have history of mumps (32.4% vs 53.1%, *P* = .011) and rheumatic fever (1.1% vs 4.4%, *P* = .028), but reported higher vaccination frequency to chicken pox (38% vs 28.1%), measles (66.5% vs 39.3%), mumps (58.7% vs 34.6%), rubella (55.3% vs 32.7%), pertussis (59.8% vs 40.1%) and pneumococcus (47.2% VS 39.4%) (*P* < .002).

**Conclusions:**

Environmental factors are important in AIH pathogenesis. Replication of these findings and prospective examination may provide new insight into AIH onset and outcomes.

## 1 | INTRODUCTION

Broken immune tolerance and failure of immunologic homeostasis underlying autoimmune hepatitis (AIH) pathogenesis are likely the result of environmental triggers in the background of a permissive genetic architecture. Similar to other autoimmune diseases, the Human Leucocyte Antigen (HLA) locus^1^ at 6p21 has been associated with AIH in prior candidate gene studies and confirmed by the only genome-wide association study.^2^ A single causal variant does not exist, and further complicating the HLA-AIH association is the presence of alleles conferring disease risk and protection.^3^ In addition to genetic factors, environmental triggers such as viral infections and drug exposures have been temporally associated with AIH onset.^4^

For other autoimmune diseases, liver diseases, primary sclerosing cholangitis (PSC) and primary biliary cholangitis (PBC), environmental risk assessments have provided a more complete understanding of disease risk, onset and possible modifiable factors.^5–7^ To date, such assessments in AIH are incomplete. Case series and small retrospective studies have linked a variety of drugs, specifically nitrofurantoin and minocycline,^8^ and viruses such as Epstein-Barr virus, varicella zoster virus, viral hepatitis A, B and C, to AIH.^4^ Unfortunately, these potentially important triggers are seldom considered systematically in clinical practice. Further evidence supports an environmental role in AIH pathogenesis, as factors such as microbiome diversity^9^ and psychosocial stress^10^ have also been associated with disease.

In this study, we aimed to identify associations between AIH and lifetime environmental exposures as well as lifestyles, personal and family histories using the Genetic Repository of Autoimmune Liver Diseases and Contributing Exposures (GRACE) study.

## 2 | METHODS

### 2.1 | AIH cases and controls

The GRACE study contains consecutively recruited AIH patients (cases) from Indiana University.^1,11^ Details regarding the establishment of this registry have been published previously. The GRACE study was developed in 2014 to collect well-phenotyped AIH cases to pursue investigation into genetic and environmental contribution to disease development. The diagnosis of AIH among cases was based on clinically accepted criteria as per the recent clinical guidelines.^12^

Controls were serially recruited from the Mayo Clinic Division of General Internal Medicine during annual visits for preventive medical examination. Control exclusion criteria included evidence of prior or current liver disease (known alcohol, fatty liver or viral hepatitis (per clinical notes, imaging or serology)) or any historical abnormal liver enzyme levels. All participants in the study had informed consent collected by study staff. This study included cohorts that adhere to the ethical guidelines of the 1975 Declaration of Helsinki, and was approved by the Institutional Review Board of Indiana University and Mayo Clinic.

### 2.2 | Questionnaire establishment and collection

The study questionnaire was developed by the Mayo Clinic Survey Centre and data acquired via this instrument among other autoimmune liver disease cohorts have been published previously.^5,6^ The survey contains questions regarding demographics, anthropometric features, education, lifestyle and environmental exposures as well as extensive personal and familial medical history. In total, the survey includes 70 primary and 300 secondary questions covering 37 pages with responses provided using scantron technology (Table S1). This study instrument was either given directly to or mailed to enrolled participants along with a prepaid return envelope. In the case of study participants (cases and controls) who did not return the first questionnaire, a second questionnaire was mailed within 2 months from the initial contact. At the time of study completion, the GRACE study included 512 AIH patients and 358 (70% response rate) had returned the study questionnaire. The control group included 563 participants (87% response rate).

### 2.3 | Statistical analysis

Information collected by the questionnaires was double entered into a SAS database. Each question was summarized by descriptive statistics for both AIH cases and controls. The *P*-values were calculated using logistic regression models, adjusting for age, gender and education. Statistical analysis was conducted in R version 3.6.2 (R Foundation for Statistical Computing) and a *P*-value of .05 was considered significant.

## 3 | RESULTS

### 3.1 | Demographics

Demographic comparison of AIH cases and controls is shown in Table 1. Cases were more likely to be female (79.3%, *P* = .045), younger at study completion (median = 55 yrs (years) old, (43 (quartile 1(Q1)), 64 (quartile 3(Q3)), *P* < .001)), less likely Caucasian (96.2%, *P* < .001) and have more college (60.3%) but less professional educational training beyond college (19.1%, *P* <.001) than healthy controls. Race remained statistically different between cases and controls after adjustment for age of recruitment, gender and level of education.

### 3.2 | Medical history: autoimmune and other conditions

AIH cases were more likely to have any another autoimmune disease in addition to AIH compared to controls (22.3% vs 10.1%, *P* < .001) (Table 2). The most common autoimmune disease found in cases in addition to AIH was autoimmune thyroid disease (17.4%), followed by rheumatoid arthritis (RA) (11.8%), Sjogren’s syndrome (5.6%), Raynaud’s syndrome (5.6%) and celiac disease (4.1%). Cases were more likely to have any autoimmune disease assessed compared to controls except for autoimmune thyroid disease, Raynaud’s syndrome and type 1 diabetes mellitus (T1DM).

History of ever having a urinary tract infection (UTI) was more frequently observed in cases compared to controls (53.6% vs 33.9%, *P* < .001), as was having recurrent UTIs (23.5% vs 15.9%, *P* = .002). Other medical conditions were more common in AIH cases as well, including symptomatic gastroesophageal reflux disease (40.4% vs 27.9%, *P* < .001), shingles (15.4% vs 12.6%, *P* = .04) and history of gallstones (20.9% vs 15.6%, *P* = .014) compared to cases. Frequencies of other assessed medical conditions did not differ the two groups (Table 2).

### 3.3 | Gynaecologic and reproductive history

Reproductive history was completed by 284 AIH cases and 414 controls (Table 3). Age at study completion was lower in cases compared to controls (55 yrs (44, 63) vs 61.2 yrs (54.3, 67.4)). There was no difference in the age of first menarche between the study groups, nor when age when menstruation stopped. AIH cases were similar to cases in regards to ever being pregnant and total live births, but had less lifetime pregnancies (1 (1, 2) vs 3 (3, 4), *P* < .001) compared to controls (Table 3).

AIH cases were more likely to have ever used oral contraceptives (OC) (83% vs 73.7%, *P* = .006) compared to controls, but the duration of OC use between groups was similar (Table 3). Among those that used OC, the age of OC start was significantly younger in AIH cases compared to controls (19 yrs (18, 22) vs 21 yrs (21, 25), *P* < .001). Among cases with a history of OC use, 93.4% were exposed to OC prior to AIH diagnosis. Ever use of hormone replacement therapy (HRT) was lower in cases compared to controls (28.5% vs 60.1%, *P* < .001). Among those that used HRT, AIH cases tended to be younger at HRT start compared to controls (45.5 yrs (37.2, 50) vs 49 yrs (44, 52), *P* = .001). Among cases with a history of HRT use, and 91.1% were exposed to HRT prior to AIH diagnosis.

### 3.4 | Health and lifestyle exposures

AIH cases were not any more likely to have every regularly smoked cigarette as controls (38.4% vs 41.2%, *P* = .888) (Table 4). Among those that had smoked, the age started smoking and years smoked regularly were no different between cases and controls. AIH cases were less likely to have been exposed to second-hand smoke at work (35.2% vs 51.6%, *P* < .001) but not at home (62.1% vs 69.2%, *P* = .748). However, AIH cases were more likely than controls to be current regular smokers (18.9% vs 7.4%, *P* = .022). AIH cases were less likely than controls to be current drinkers (27.7% vs 76.7%, *P* < .001). Among individuals that ever consumed alcohol, AIH cases had less duration of consumption (27 years (12, 37) vs 40 (31, 47), *P* < .001) compared to controls.

### 3.5 | Childhood diseases and immunizations

AIH cases were less likely to have the infectious childhood disease mumps (32.4% vs 53.1%, *P* = .011) and rheumatic fever (1.1% vs 4.4%, *P* = .028) compared to controls (Table 5). There was no difference between study groups according to the frequency of chicken pox, measles or rubella. Assessment of vaccination history revealed that AIH cases more frequently had vaccination to chicken pox (38% vs 28.1%, *P* = .005), measles (66.5% vs 39.3%, *P* < .001), mumps (58.7% vs 34.6%, *P* < .001), rubella (55.3% vs 32.7%, *P* < .001), pertussis (59.8% vs 40.1%, *P* < .001) and pneumococcus (47.2% vs 39.4%, *P* = .002) (Table 5).

### 3.6 | Family history

Detailed history of autoimmune diseases, gastrointestinal and liver conditions among both first-degree and second-degree relatives (FDRs and SDRs) was collected in cases and in controls (Table S2). The AIH case pedigrees included 2,126 FDRs and 4,068 SDRs. Controls had 3,753 FDRs and 5,210 SDRs pedigrees available. AIH cases of FDRs were more likely to have celiac disease (3.6%, *P* = .001), lupus (3.9%, *P* = .028), multiple sclerosis (3.9%, *P* < .001), RA (13.4%, *P* < .001), Sjogren’s syndrome (2.8%, *P* < .001), T1DM (10.6%, *P* < .001) and colon cancer (10.3%, *P* < .001) compared to control FDRs. AIH cases of SDRs were more likely to have RA (6.7%, *P* < .001) and T1DM (5%, *P* < .001) compared to control SDRs.

### 3.7 | Environmental risk discrepancy according to gender

Prior disparities among genders in AIH studies have revealed that male patients have higher frequencies of DRB1*03:01, worse overall survival and increased development of hepatocellular cancer.^13–15^ Thus, stratification of reported medical histories and exposures were completed according to gender. Observed differences between medical histories in cases and controls were most often associated with female gender (Table S3).

## 4 | DISCUSSION

In this study, patients with AIH compared to controls were more likely to report history of UTI, recurrent UTIs, gallstones, multiple vaccinations (chicken pox, measles, mumps, rubella, pertussis and pneumococcal), younger age of OC start, ever OC use and current smoking. Moreover, AIH cases were less likely to report a history of working with a smoker, mumps, rheumatic fever, pregnancies and HRT. Finally, patients with AIH often reported concurrent autoimmune diseases (including celiac disease, RA, and Sjogren’s syndrome) as well as FDR with autoimmune diseases (celiac disease, MS, RA, SS, T1DM) as well as SDR (RA, T1DM).

The GRACE study is the largest cohort of AIH cases to date that has systematically evaluated environmental risk factors associated with the development of disease, but observations in this study should still be interpreted in the context of the study design. Significant differences between case and control responses in this study do not necessarily indicate disease causation, but must be interpreted in the hypothetical web of interactions (environment and genetics) that underscore disease development. Despite a systematic approach in this study, we also realize that there are many unmeasured environmental exposures starting at conception. However, we utilized a questionnaire that has been implemented in other autoimmune liver disease cohorts and collected environmental exposures that have either biologic plausibility in autoimmunity or have been associated with other chronic diseases. To our knowledge, only one other study^16^ has assessed a diverse array of environmental risk factors in AIH. Ngu *et al* reported the only factor that independently increased AIH risk was exposure to antibiotics within 12 months of diagnosis among 72 AIH cases.^16^ This study also observed that alcohol and having a childhood home with wood heating was associated with reduced risk of AIH development. Unfortunately, our survey instrument did not assess drug exposure history, but we suspect this finding from the Ngu *et al* study could represent an enrichment of drug-induced AIH. Mechanistically, this likely represents a subtype of AIH as immunosuppression removal can be completed in a high proportion of patients^8^ and has been shown to have differing genetic risks than idiopathic AIH.^17^ Our findings of alcohol use in our cohort were supportive of a prior observation,^18^ as we observed AIH cases were less likely to be current drinkers and had less alcohol duration on average than controls. Alcohol has been shown to be protective against progression of other autoimmune diseases (ie SLE^19^ and RA^20^) and may be secondary to impaired presentation of foreign antigen by antigen-presenting cells.^21^ Despite a possible role of protection in AIH, alcohol use beyond diagnosis may be very different. Mortality hazard ratio has been shown higher in alcoholic AIH patients versus those without alcohol history, thus clinical screening for alcohol use and expressed alcohol avoidance remain important.^18^

Smoking has not been assessed previously in AIH patients, yet it has been well documented in other autoimmune liver diseases PSC and PBC. In PSC, smoking most recently has been shown to only associate with disease among patients that also had concurrent IBD.^5^ On the other hand, PBC has routinely been associated with smoking.^6,22–24^ In our study, we observed no difference in AIH cases compared to controls among frequency of “ever regularly smoked”, age of smoking start and years of regularly smoked. However, we did observe that AIH cases were more likely to currently smoke (18.9% vs 7.4%, adjusted *P*-value =.022) but less likely to ever work with a smoker (35.2% vs 51.6%, adjusted *P*-value <.001). These conflicting results may have multiple explanations that could include a dose effect of smoking, variable known/unknown genetic predispositions in our cohort or highlight the tenuous balance between pro- and anti-inflammatory attributes of smoking among populations. Even despite the relative consistency of smoking associated with PBC in prior studies, published cohorts have shown conflicting assessments.^25,26^ Cigarette smoking has a diverse immunomodulatory effect and includes increases in proinflammatory cytokines and tumour necrosis factor alpha^27^ as well as decreased activation of antigen-presenting cells with diminished Th1 and Th17 polarizing (reduced IL-12 and IL-23) effect in the setting of lipopolysaccharide.^28^ AIH patients that smoked prior to diagnosis reported a median of 17.2 pack-years, whereas in a prior PBC study,^6^ PBC cases had a median of 10.5 pack-years. Beyond clarity of impact on risk of disease development, AIH further lacks investigation into impact of smoking on disease and fibrosis progression and remains an important research question. This remains particularly important, as nearly 20% of our AIH cohort was smoking at time of survey completion.

Notably, a novel and possibly most important finding in this study was the observation of increased UTI diagnosis (53.6% vs 33.9%) and recurrent UTIs (23.5% vs 15.9%) among AIH cases versus controls (Table 2). This observation in PBC was first made in 1984^29^ and has been observed prior to diagnosis, thus less likely a result of predisposing risk from autoimmune disease. Similarly, PSC cases have reported higher frequencies of recurrent UTI compared to controls in a prior study with mechanistic uncertainty,^5^ but may be a result of molecular mimicry of bacterial antigens and host proteins.^30^ Certainly PBC and PSC are very different diseases than AIH, as both are resultant of dysregulated inflammatory response associated with small to large bile ducts as opposed to interface and lobular inflammation. Reconciliation of observed increased “ever UTI” and recurrent UTI in AIH cases compared to controls is capable when considering PBC, as both diseases have female predominance with similar age medians of onset. Yet, not true for PSC, as often a male dominant disease of young onset. Risk factor stratification according to gender supports the gender-specific risk observation, as the risk of UTI and recurrent UTI was only associated with female cases in this study (Table S2). Increased “ever UTI” and recurrent UTIs in AIH may be a surrogate for other underlying aetiology such as antibiotic use, fitting with the Ling et al study.^16^ Furthermore, drug-induced AIH is well described, and likely represents a subtype of rather heterogenous AIH populations. Finally, one should consider that both PBC^31^ and AIH^4^ have shown increased frequency of other historical infections (Epstein-Barr virus, CMV), and may suggest a role for multiple infections (with risk for molecular mimicry) in autoimmune liver disease development.^32,33^

Given female predominance of AIH, we must carefully consider the results of documented exposures that correlate with gender. Our data revealed that AIH cases were more likely to ever use OC, start OC younger (but similar duration), have fewer lifetime pregnancies and less likely to have ever used HRT compared to controls. The observed difference in AIH protection (HRT) and risk (OC use) observed between these oestrogen containing pharmaceuticals may relate to the inherent timing of administration. Infrequent HRT use may be driven by behaviours of cautious providers aware of patients known liver disease (average age AIH diagnosed 50 years old (37, 58)). Similar observations (less HRT use) have been observed in females with PSC,^5^ yet in PBC, HRT and OC use have been associated with disease protection.^34^ Generally speaking, oestrogens are associated with known anti-inflammatory properties via multiple mechanisms.^35^ Sex hormones are also important in the complex development of both the innate and adaptive immune system^36^ and have been associated with protection among other autoimmune diseases such as RA and MS.^37^ Further examination of hormone-related effects on AIH development and disease progression may illuminate key pathogenetic differences among autoimmune liver diseases.

Historically, only in few, historical and rare cases, has autoimmune pathology been firmly linked with particular vaccines. An observed polyradiculoneuritis associated with the 1976–1977 vaccination campaign against swine influenza is one such example.^38^ We report increased prevalence of chicken pox, measles, mumps, rubella, pertussis and pneumococcal vaccination among AIH cases compared to controls. We also observed less infectious reports of mumps and rheumatic fever in AIH cases. Vaccines are inherently immunogenic, shaping a host immunologic response to a focused antigen that remains controlled. Prior reports of vaccination association with AIH have been mostly case based,^39,40^ without strong evidence linking current vaccine protocols to increased disease development. However, we believe further investigation of vaccines as a prospective focus may help to elucidate a clear association with autoimmune liver disease. Furthermore, vaccine-related AIH risk was associated with female gender, except for pertussis vaccine. These observed risks may highlight fundamental disease differences in pathogenesis between males and females including other gender-specific risks and interactions (increased HLA-DRB1*03:01 in males^13,14^ and hormonal therapy or pregnancy in females).

We admit that given the observational approach to our study, there are limitations worth considering. Despite being one of the first systematic approaches to examine environmental risk in well-phenotyped AIH case development, the size of our study remains moderate. We were dependent on patient-reported data for many outcomes of interest which may introduce recall bias. Furthermore, this case-control study does not include a disease control group for direct comparison, yet two other liver disease populations (PBC and PSC) have utilized this questionnaire previously.^5,6^ We were careful to compare our findings to these prior studies, yet given the inherent disease differences, we must consider our observations in the context of AIH. This methodology is necessary until well-cultivated prospective studies of AIH are established and completed. We believe our cohort is an excellent representation of AIH patients and well poised to address these questions, as other key findings support prior observations of similar demographics, concurrent autoimmune disease and enrichment of FDR (and few SDR) with autoimmune diseases (Table S2).

In summary, we report novel associations between a variety of exogenous risk factors and AIH using an established AIH cohort (GRACE study) and healthy controls. History of UTI, recurrent UTI, ever and earlier start of OC, current smoking and a variety of immunizations were associated with the development of AIH. On the other hand, history of mumps, HRT use, history of working with a smoker were protective against AIH. Despite plausible biologic mechanisms, these associations do not indicate disease causation. Yet, counselling on smoking cessation, screening for other autoimmune illnesses at diagnosis and during follow-up and careful antibiotic stewardship remain important and are currently actionable by clinicians. We advocate for multicentre prospective studies of AIH to better understand modifiable risk factors in the setting of a permissive genetic framework.

## Supplementary Material

Supplementary Material

## Figures and Tables

**TABLE 1:** Demographics of autoimmune hepatitis cases and controls

	Mayo Controls (N = 563)	AIH Cases (N = 358)	*P*-value*
Female, %	414 (73.5%)	284 (79.3%)	.045
Race (Caucasian), %	557 (99.3%)	327 (96.2%)	<.001
Age at diagnosis^a^, yrs	N/A	50 (37, 59)	
Median age at recruitment^a^, yrs	61.2 (54.1, 68.0)	55.0 (43.0, 64.0)	<.001
Education			<.001
Some High School	106 (19.0%)	72 (20.6%)	
College	275 (49.3%)	211 (60.3%)	
Professional Training Beyond College	177 (31.7%)	67 (19.1%)	

Abbreviation: Yrs, years.

a variable summarized as median (1st quartile, 3rd quartile).

* unadjusted *P*-values.

**TABLE 2:** Autoimmune disease and other medical history in autoimmune hepatitis cases and controls

	Mayo Controls (N = 563)	AIH Cases (N = 358)	*P*-value*
Autoimmune Diseases			
Celiac disease	4 (0.7%)	14 (4.1%)	.017
Raynaud’s syndrome	24 (4.3%)	19 (5.6%)	.31
RA	29 (5.2%)	40 (11.8%)	<.001
Sjogren’s syndrome	6 (1.1%)	19 (5.6%)	<.001
T1DM	2 (0.4%)	4 (1.2%)	.86
Thyroid disease	100 (17.8%)	59 (17.4%)	.86
Any Autoimmune Disease (not AIH)	57 (10%)	76 (22.3%)	<.001
Other Medical Conditions			
GERD	157 (27.9%)	137 (40.4%)	<.001
Gall stones	88 (15.6%)	70 (20.9%)	.014
Myocardial infarction	15 (2.7%)	5 (1.5%)	.80
Shingles	71 (12.6%)	52 (15.4%)	.040
UTI	191 (33.9%)	181 (53.6%)	<.001
Recurrent UTI	89 (15.9%)	81 (23.5%)	.006
Number of recurrent UTIs			.35
Once every 2 years	33 (38.8%)	21 (25.9%)	
Once every year	18 (21.2%)	24 (29.6%)	
Two or more times a year	34 (40.0%)	36 (44.4%)	

Abbreviations: AIH, autoimmune hepatitis; RA, rheumatoid arthritis; T1DM, Type 1 diabetes mellitus; UTI, urinary tract infection.

* *P*-values are adjusted for age at time of recruitment, gender and education based on logistic regression analysis.

**TABLE 3:** Gynaecologic and reproductive history of female autoimmune hepatitis cases and controls

	Mayo Controls (N = 414)	AIH Cases (N = 284)	*P*-value*
Age at recruitment, yrs^a^	61.2 (54.3, 67.4)	55 (44, 63)	–
Education			
Some high school	81 (19.8%)	55 (19.9%)	–
College	217 (52.9%)	173 (62.5%)	
Professional training beyond college	112 (27.3%)	49 (17.7%)	
Age first menarche			
Less than 12 yrs	65 (18.2%)	46 (17.4%)	.18
12 yrs	97 (27.2%)	65 (24.5%)	
13 yrs	98 (27.5%)	69 (26.0%)	
14 yrs	53 (14.8%)	42 (15.8%)	
15 yrs or older	44 (12.3%)	43 (16.2%)	
Age menstruation ceased, yrs^a^	N = 306, 49 (43, 52)	N = 161, 46 (41, 50)	.08
Ever pregnant	356 (86.4%)	220 (79.1%)	.34
Number of pregnancies^a^	3 (2, 4)	1 (1,2)	<.001
Number of live births^a^	2 (2,3)	1 (1,2)	.07
Age first pregnancy			
17 yrs or younger	27 (7.6%)	21 (9.7%)	.08
18 yrs	21 (5.9%)	8 (3.7%)	
19 yrs	27 (7.6%)	18 (8.3%)	
20 to 24 yrs	161 (45.2%)	74 (34.1%)	
25 to 29 yrs	85 (23.9%)	54 (24.9%)	
30 to 34 yrs	27 (7.6%)	30 (13.8%)	
35 to 39 yrs	8 (2.2%)	12 (5.5%)	
40 yrs or older	0 (0.0%)	0 (0.0%)	
Pruritus during pregnancy	301 (94.1%)	163 (88.1%)	.29
Ever oral contraceptives (OC)	302 (73.7%)	230 (83.0%)	.006
Age OC start, yrs^a^	21 (19, 25)	19 (18, 22)	<.001
Years taking OC			
Less than 1 yrs	29 (9.8%)	23 (10.0%)	.73
1 to 5 yrs	118 (39.9%)	80 (34.9%)	
6 to 10 yrs	71 (24%)	64 (27.9%)	
11 yrs or more	78 (26.4%)	62 (27.1%)	
Hormone replacement therapy (HRT) ever	247 (60.1%)	79 (28.5%)	<.001
Age HRT Start, yrs^a^	49 (44, 52)	45.5 (37.2, 50)	.001

Abbreviations: AIH, autoimmune hepatitis; HRT, hormone replacement therapy; OC, oral contraceptives; yrs, years.

a variables summarized as median (Q1, Q3).

* *P*-values are adjusted for age at time of recruitment and education based on logistic regression analysis.

**TABLE 4:** Smoking and alcohol history of AIH cases and controls

	Mayo Controls (N = 563)	AIH Cases (N = 358)	*P*-value*
Tobacco exposure			
Every regularly smoked	231 (41.2%)	134 (38.4%)	.89
Age started smoking, yrs^a^	18 (16, 20)	18 (16, 20)	.80
Currently smoke regularly	17 (7.4%)	24 (18.9%)	.022
Smoked regularly, yrs^a^	17 (9, 29)	19.5 (8, 32)	.13
Ever lived with smoker	389 (69.2%)	216 (62.1%)	.75
Ever worked with smoker	288 (51.6%)	122 (35.2%)	.001
Ever lived or worked with smoker	449 (79.9%)	246 (70.7%)	.17
Pack yrs prior AIH diagnosis	NA	17.2 (9.6, 26.4)	
Alcohol exposure			
Currently drink alcohol	76.30%	27.70%	<.001
Average alcoholic drinks per month			
1 to 10 drinks per month	200 (46.8%)	34 (50.0%)	.32
11 to 20 drinks per month	76 (17.8%)	16 (23.5%)	
21 to 30 drinks per month	75 (17.6%)	9 (13.2%)	
31 to 40 drinks per month	39 (9.1%)	3 (4.4%)	
41 to 50 drinks per month	15 (3.5%)	6 (8.8%)	
51 or more drinks per month	22 (5.2%)	0 (0.0%)	
Yrs drank alcohol^a^	40 (31, 47)	27 (12, 37)	<.001

Abbreviation: Yrs, years.

a variables summarized as median (Q1, Q3).

* *P*-values are adjusted for age at time of recruitment and education based on logistic regression analysis.

**TABLE 5:** Childhood disease and immunization history in AIH Cases and Controls

	Mayo Controls (N = 563)	AIH Cases (N = 358)	*P*-value*
Childhood diseases			
Chicken Pox	399 (70.9%)	258 (72.1%)	.09
Measles	320 (56.8%)	134 (37.4%)	.12
Mumps	299 (53.1%)	116 (32.4%)	.011
Rheumatic Fever	25 (4.4%)	4 (1.1%)	.028
Vaccination history			
Chicken Pox	158 (28.1%)	136 (38.0%)	.005
Influenza	426 (75.7%)	276 (77.1%)	.23
Measles	221 (39.3%)	238 (66.5%)	<.001
Mumps	195 (34.6%)	210 (58.7%)	<.001
Rubella	184 (32.7%)	198 (55.3%)	<.001
Small Pox	326 (57.9%)	209 (58.4%)	.11
Pertussis	226 (40.1%)	214 (59.8%)	<.001
Pneumococcal	222 (39.4%)	169 (47.2%)	.002
Polio	452 (80.3%)	292 (73.2%)	.38

* *P*-values are adjusted for age at time of recruitment and education based on logistic regression analysis.

## Data Availability

The data that support the findings of this study are available from the corresponding author upon reasonable request.

## Abbreviations:

AIH: Autoimmune Hepatitis
GRACE: Genetic Repository of Autoimmune Liver Disease and Contributing Exposures
PBC: Primary Biliary Cholangitis
PSC: Primary Sclerosing Cholangitis
UC: Ulcerative Colitis
