# Supplementary material for: Environmental risk factors are associated with autoimmune hepatitis
Source: Liver Int. 2021 May 28;41(10):2396–403. doi: 10.1111/liv.14944 (PMC8496440; doi:10.1111/liv.14944)
Supplement: Supplementary file 1 — Table S1‐3 [file LIV-41-2396-s001.docx]

**Supplemental Table 1: Environmental Questionnaire**

| **Environmental Questionnaire** |
| --- |
| Date of birth |
| Describe health during last year (excellent, very good, good, fair, poor) |
| Sex |
| Current height & weight |
| Hispanic/Latino |
| Asian, Black/African American, White, Native American/Alaska Native, Native Hawaiian/Pacific Islander, other |
| Highest grade/level of education completed |
| During the past 12 months, which vitamins, minerals, or supplements have you taken regularly (2x week or more for 3 months) |
| Current medications |
| Past Immunizations |
| Business or Industry worked for majority of life |
| Exposure to environmental toxins |
| Primary residence, birth to 18 years old |
| Current residence |
| Where have you traveled (not including where you have lived) |
| For job held the longest, how much time did you engage in sitting, standing, walking, light manual labor, heavy manual labor |
| Consumption of caffeinated coffee, tea, or soda |
| How many years have you drank caffeinated coffee, tea, or soda |
| Lifetime consumption of unpasteurized milk |
| Ever consumed alcoholic beverages on regular basis (at least once per month) |
| Used any of the following tobacco products for 12 months or longer |
| Ever smoked at least 100 cigarettes in lifetime |
| Did you ever work where others smoked regularly |
| Current relationship status and length in years |
| Indicate the age you were first diagnosed with the following conditions: arthritis, ankylosing spondylitis, autoimmune thyroid disease, lupus, multiple sclerosis, psoriasis, Raynaud’s phenomenon, scleroderma, Sjogren syndrome, PSC, PBC, alcoholic liver disease, gallstones, autoimmune hepatitis, hepatitis A/B/C, other liver disease, UTI, measles, mumps, chicken pox, shingles, rheumatic fever, breast cancer, pancreatic cancer, stomach cancer, color/rectal cancer, liver cancer, uterine/endometrial cancer, cervical cancer, prostate cancer, heart attack myocardial infarction, high cholesterol, blood clots, acid reflux/GERD, celiac disease, Crohn’s disease, ulcerative colitis, type 1 diabetes, type 2 diabetes |
| Did/do you have recurrent episodes of UTI |
| Liver biopsy completed |
| Recipient of liver transplant |
| Other transplant |
| Number of: full sisters, full brothers, half-sisters, half-brothers, stepsisters, stepbrothers, blood-related daughters, blood-related sons, adopted daughters, adopted sons, maternal aunt, maternal uncle, maternal first cousins, paternal aunts, uncles, first cousins |
| **Women only:** |
| Age when menstrual periods began |
| Uterus removed/last menstrual period more than 12 months ago |
| Surgery to remove one or both ovaries |
| Ever pregnant |
| Ever used birth control pills, patches, implants, or shots |
| Hormone replacement used other than birth control pills |
| **Family History:** |
| Year of birth |
| Living/Deceased |
| Current age or age of death |
| If deceased, cause of death |
| Age diagnosed with following conditions: PBC, PSC, liver cancer, Crohn’s disease, ulcerative colitis, autoimmune thyroid disease, Sjogren syndrome, celiac disease, rheumatoid arthritis, lupus, type 1 diabetes, MS, gallstones, colon/rectal cancer, other cancer |

**Supplemental Table 2: Family History (First- and Second-Degree Relatives) of Autoimmune Diseases and Medical Conditions among AIH Cases and Controls**

|  | Mayo Controls (N=563) | AIH Cases (N=358) | p-value |
| --- | --- | --- | --- |
| Celiac: FDR | 4 (0.7%) | 13 (3.6%) | 0.001 |
| Celiac: SDR | 1 (0.2%) | 3 (0.8%) | 0.14 |
| Colon Cancer: FDR | 17 (3.0%) | 37 (10.3%) | <0.001 |
| Colon Cancer: SDR | 77 (13.7%) | 12 (3.4%) | <0.001 |
| Crohn’s: FDR | 21 (3.7%) | 9 (2.5%) | 0.31 |
| Crohn’s: SDR | 5 (0.9%) | 4 (1.1%) | 0.73 |
| Liver Cancer: FDR | 34 (6.0%) | 19 (5.3%) | 0.64 |
| Liver Cancer: SDR | 28 (5.0%) | 12 (3.4%) | 0.24 |
| Lupus: FDR | 9 (1.6%) | 14 (3.9%) | 0.028 |
| Lupus: SDR | 2 (0.4%) | 3 (0.8%) | 0.33 |
| Multiple Sclerosis: FDR | 4 (0.7%) | 14 (3.9%) | <0.001 |
| Multiple Sclerosis: SDR | 4 (0.7%) | 3 (0.8%) | 0.83 |
| PBC: FDR | 6 (1.1%) | 9 (2.5%) | 0.09 |
| PBC: SDR | 3 (0.5%) | 5 (1.4%) | 0.17 |
| PSC: FDR | 3 (0.5%) | 3 (0.8%) | 0.58 |
| PSC: SDR | 0 (0.0%) | 2 (0.6%) | 0.08 |
| RA: FDR | 11 (2.0%) | 48 (13.4%) | <0.001 |
| RA: SDR | 5 (0.9%) | 24 (6.7%) | <0.001 |
| Sjogren’s: FDR | 0 (0%) | 10 (2.8%) | <0.001 |
| Sjogren’s: SDR | 0 (0.0%) | 2 (0.6%) | 0.08 |
| Thyroid Disease: FDR | 121 (21.5%) | 65 (18.2%) | 0.22 |
| Thyroid Disease: SDR | 27 (4.8%) | 21 (5.9%) | 0.48 |
| T1DM: FDR | 9 (1.6%) | 38 (10.6%) | <0.001 |
| T1DM: SDR | 4 (0.7%) | 18 (5.0%) | <0.001 |
| Ulcerative Colitis: FDR | 29 (5.2%) | 22 (6.1%) | 0.52 |
| Ulcerative Colitis: SDR | 13 (2.3%) | 10 (2.8%) | 0.65 |

FDR: first degree relative, SDR: second degree relative, PBC: primary biliary cholangitis, PSC: primary sclerosing cholangitis, RA: rheumatoid arthritis, T1DM: Type 1 diabetes mellitus

p-values are not adjusted in this table

**Supplemental Table 3: Stratification of Medical History and Exposures According to Gender**

|  | Adjusted Odds Ratios (95% Confidence Interval) * | | |
| --- | --- | --- | --- |
| Risk Factor | AIH Cases vs. Controls | Female AIH Cases vs. Controls | Male AIH Cases vs. Controls |
| *Medical history*  Celiac disease | 4.38 (1.48 - 16.72) | 3.43 (0.92, 16.48) | -- |
| Rheumatoid arthritis | 2.62 (1.55 - 4.48) | 2.82 (1.61 - 4.99) | -- |
| Sjogren's Syndrome | 5.77 (2.31 - 16.44) | 5.8 (2.32 - 16.58) | -- |
| Any autoimmune disease beyond AIH | 2.48 (1.66 - 3.72) | 2.63 (1.70 - 4.07) | 1.73 (0.55 - 5.07) |
| GERD | 2.05 (1.51 - 2.78) | 2.22 (1.57 - 3.15) | 1.51 (0.76 - 2.93) |
| Gall Stones | 1.59 (1.10 - 2.31) | 1.50 (1.01 - 2.23) | 2.75 (0.90 - 8.45) |
| Shingles | 1.53 (1.02 - 2.29) | 1.65 (1.06 - 2.59) | 0.98 (0.33 - 2.60) |
| UTI | 2.45 (1.79 - 3.38) | 2.79 (1.98 - 3.95) | 0.86 (0.28 - 2.37) |
| Recurrent UTI | 1.68 (1.16 - 2.43) | 1.76 (1.21 - 3.97) | -- |
| Mumps | 0.67 (0.49 - 0.91) | 0.63 (0.44 - 0.89) | 0.88 (0.45 - 0.72) |
| Rheumatic Fever | 0.30 (0.09 - 0.79) | 0.38 (0.11 - 1.03) | -- |
| Measles vaccination | 2.67 (1.96 - 3.64) | 3.33 (2.30 - 4.86) | 1.54 (0.84 - 2.84) |
| Mumps vaccination | 2.06 (1.52 - 2.81) | 2.35 (1.64 - 3.39) | 1.44 (0.77 - 2.66) |
| Rubella vaccination | 1.90 (1.39 - 2.60) | 1.96 (1.38 - 2.81) | 1.70 (0.88 - 3.28) |
| Pertussis vaccination | 2.03 (1.51 - 2.74) | 1.78 (1.27 - 2.51) | 3.16 (1.66 - 6.02) |
| Pneumococcal vaccination | 1.61 (1.18 - 2.19) | 1.53 (1.08 - 2.16) | 0.91 (0.48 - 1.78) |
| *Lifestyle*  Currently smoke regularly | 2.3 (1.13 - 4.72) | 1.22 (0.54 - 2.74) | 29.47 (5.33 – 31.8) |
| Ever worked with a smoker | 0.61 (0.45 - 0.82) | 0.69 (0.49 - 0.97) | 0.38 (0.20 - 0.72) |
| Currently drink alcohol | 0.1 (0.07 - 0.16) | 0.08 (0.05 - 0.13) | 0.18 (0.08 - 0.39) |
| Years drank alcohol | 0.95 (0.93 - 0.96) | 0.95 (0.93 - 0.97) | 0.94 (0.91 - 0.97) |

NS: Not statistically significant, p > 0.05

* Odd’s ratios are adjusted for age at time of recruitment and education based on logistic regression analysis. In the subgroup analyses, OR are not shown for variables that were not present in at 5% in either the AIH cases or controls.
